# Natriuretic peptide-directed medical therapy: a systematic review

**DOI:** 10.1186/s13741-019-0134-y

**Published:** 2020-02-18

**Authors:** Christella S. Alphonsus, Pooveshnie Govender, Reitze N. Rodseth, Bruce M. Biccard

**Affiliations:** 1 Consultant Anaesthesiologist, University of Cape Town, Western Cape, South Africa; 2 Anaesthesia Fellow, The Royal Papworth Hospital, Cambridgeshire, UK; 3 Consultant Anaesthesiologist, University of KwaZulu-Natal, KwaZulu-Natal, South Africa

**Keywords:** Cardiac morbidity, Pre-operative evaluation, Myocardial ischemia

## Abstract

Natriuretic peptides (NP) are strongly associated with perioperative cardiovascular events. However, in patients with raised NP, it remains unknown whether treatment to reduce NP levels prior to surgery results in better perioperative outcomes. In this systematic review and meta-analysis, we investigate NP-directed medical therapy in non-surgical patients to provide guidance for NP-directed medical therapy in surgical patients. The protocol was registered with PROSPERO (CRD42017051468). The database search included MEDLINE (PubMed), CINAHL (EBSCO host), EMBASE (EBSCO host), ProQuest, Web of Science, and Cochrane database. The primary outcome was to determine whether NP-directed medical therapy is effective in reducing NP levels within 6 months, compared to standard of care. The secondary outcome was to determine whether reducing NP levels is associated with decreased mortality. Full texts of 18 trials were reviewed. NP-directed medical therapy showed no significant difference compared to standard care in decreasing NP levels (standardized mean difference − 0.04 (− 0.16, 0.07)), but was associated with a 6-month (relative risk (RR) 0.82 (95% confidence interval (CI) 0.68–0.99)) reduction in mortality.

## Introduction

Every year, 230 million adults undergo non-cardiac surgery worldwide (Weiser et al. 2008). In patients who are 45 years or older, 8% will suffer Myocardial Injury after Non-cardiac Surgery (MINS) (Botto et al. 2014) and 2% will die within 30 days (Devereaux et al. 2012). MINS is typically asymptomatic without the usual features of chest pain and electrocardiogram changes seen with myocardial infarction (Botto et al. 2014). MINS has prognostic importance up to a year after surgery (Puelacher et al. 2018).

The biomarker, B-type natriuretic peptide (BNP), has been identified as an important preoperative predictor of perioperative cardiovascular events (Rodseth et al. 2014). Despite this strong association, it remains unknown whether preoperative treatment to normalize or reduce NP (B-type natriuretic peptide and N-terminal pro-B-type natriuretic peptide) levels prior to surgery would result in improved perioperative outcomes. This is a novel approach that has not been tested in clinical trials involving surgical patients. Thus, a systematic review of non-surgical trials is necessary to establish whether this approach is safe and effective before it can be tested in a surgical population.

The objective of this systematic review of clinical trials was to determine whether, in adults, medical patients with cardiac failure, NP-directed medical therapy is able to decrease NP levels and whether this is associated with increased survival.

These data could then be used to inform preoperative protocols aimed at decreasing NPs prior to surgery, with the intention of improving perioperative cardiovascular outcomes.

## Methods

### Protocol and registration

The protocol was registered with PROSPERO (CRD42017051468). The Preferred Reporting Items for Systematic reviews and Meta-Analysis (PRISMA) guidelines were adhered to (Moher et al. 2009).

#### Eligibility criteria

Clinical trials of adult medical patients who were randomized to either NP guided medical therapy or standard care were eligible. We included trials which used NPs to (i) guide medical therapy in non-surgical patients, (ii) up-titrate or modify medical therapy in the response to NP levels, or (iii) included exercise as part of cardiac rehabilitation in non-surgical patients. We required that the trials report the subsequent changes in NP levels. We excluded trials that (i) monitored natriuretic peptides for prognostic or diagnostic purposes, without a strategy to lower natriuretic peptide levels, (ii) reviews of natriuretic peptide or biomarker physiology, and (iii) trials reporting natriuretic peptides in patients with acute myocardial infarction, pulmonary hypertension, cardiac resynchronization therapy, and left ventricular devices.

#### Information sources, search, and study selection

Three searches were conducted using search terms “brain natriuretic peptide” AND “treatment,” “brain natriuretic peptide” AND “heart failure” and “brain natriuretic peptide” AND “exercise.” The following databases were accessed; MEDLINE (PubMed), CINAHL (EBSCO host), EMBASE (EBSCO host), ProQuest, Web of Science, and Cochrane database. No date filter was used. An example of the search is shown in Additional file 1. The initial search was conducted on 22 December 2016 and updated on the 4 March 2018.

#### Data collection process

Titles were screened for potential inclusion by CA and PG. The abstracts of the potential papers identified through the title search were then screened using inclusion and exclusion criteria by CA and PG. The full texts of potential trials were then extracted for detailed review and analysis. Reference lists were searched for additional papers that could be included in this review. Data extraction was done by one author (CA) and then checked by a co-author. When the required data was not presented in the publication, the authors were contacted for these data.

#### Data items

We extracted data on the NP reduction within the first six months of randomization and mortality at 6 months. The data items extracted for this review are shown in Additional file 1: Table S1.

#### Outcomes

The primary outcome for this review was to determine whether a NP-directed medical therapy protocol is effective in reducing NP levels at 6 months after initiation of therapy compared to standard care. The secondary outcome was to determine whether NP-directed medical therapy decreases mortality at 6 months and at the end of the trial. The safety outcomes of changes in medical therapy were evaluated. Specific medical treatment strategies are described.

#### Risk of bias in individual studies

Assessment of bias in the studies was conducted by CA and verified by BB following discussion. Each randomized trial was assessed using the Cochrane Collaboration risk of bias tool, assessing selection bias, concealment bias, performance bias, detection bias, attrition bias, and other bias. Studies were assessed as having a low, unclear, or high risk of bias.

#### Summary measures and synthesis of results

The statistical analyses were conducted using Review Manager Version 5.3 (Copenhagen: The Nordic Cochrane Centre, The Cochrane Collaboration, 2014). Those trials which had data on NP levels within 6 months of therapy initiation were analyzed using standardized mean difference (SMD), and these data are presented as a forest plot. Mean and standard deviation (SD) of NP levels were used and those trials which reported NP levels as the median and interquartile range (IQR) were converted to mean and SD, using the formula proposed by Wan et al. (Wan et al. 2014). Reporting the SMD allowed for the inclusion of all trials, whether BNP or NT-proBNP, was used to monitor the medical therapeutic response. SMD addresses the difference in the effect size for an intervention when the units of measurement differ between trials e.g. use of BNP or NT-proBNP. The SMD is the difference between groups in the mean endpoint divided by the SD of the control group or pooled SD of the treatment and control groups (Hedges’ *g*) (Guyatt et al. 2015).

A meta-analysis of mortality within 6 months of the initiation of therapy, with subgroup analyses at 4 and 6 months was conducted. The results are reported as relative risk (RR), with 95% confidence intervals (CI), and presented as forest plots. Random effects models were used where the *I*^2^ statistic > 2s5% (representing significant heterogeneity), otherwise a fixed-effects model was used.

#### Risk of bias across studies

Risk of bias across studies was assessed with funnel plots for NP reduction and mortality.

#### Post hoc

After extracting and analyzing the data, it was noticed that the methodology used in the exercise trials differed significantly from the medical therapy trials. This difference was so substantial that we deemed it inappropriate to pool the two interventions. We therefore made a post hoc decision to separate the exercise studies from the medical therapy studies. These exercise study data are presented in the accompanying publication (CS Alphonsus et al. 2019).

## Results

### Study selection

**Fig. 1:**
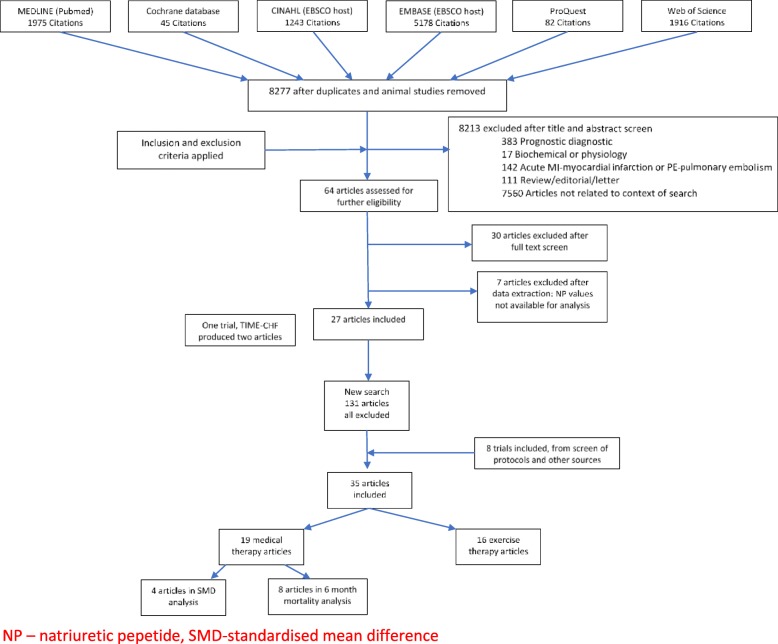
PRISMA flow diagram

Sixty-four full-text articles were reviewed for potential inclusion and 26 trials (presented in 27 publications) met the inclusion criteria. An additional eight trials were added from references (Fig. 1). Eighteen trials of medical therapy interventions were identified (reported in 19 publications; 1 trial was reported in 2 separate papers) (Maeder et al. 2013; Pfisterer et al. 2009) fulfilled the inclusion criteria, although only 14 trials provided data for this review’s outcomes. The 16 exercise trials were subsequently removed from this review, following the post hoc decision to present these trials in a separate paper (CS Alphonsus et. al. 2019).

We evaluated previous systematic reviews identified in the search using the AMSTAR format (Additional file 1: Table S2).

### Study characteristics of included studies

The characteristics of the included clinical trials are shown in Table 1. These trials included adult patients of 18 years and older. The majority of the trials examined outpatient NP-directed medical therapy, with follow-up of 15 months or more. In 10 out of 18 trials, patients were seen by a specialist at a clinic (Anguita et al. 2010; Berger et al. 2010; Eurlings et al. 2010; Januzzi et al. 2011; Jourdain et al. 2007; Lainchbury et al. 2009; Murdoch et al. 1999; Persson et al. 2010; Schou et al. 2013; Shah et al. 2011; Troughton et al. 2000). Most patients were randomized once heart failure was medically stabilized and 12 out of 18 trials enrolled patients with EF ≤ 50% (Beck-da-Silva et al. 2005; Felker et al. 2017; Januzzi et al. 2011; Jourdain et al. 2007; Karlstrom et al. 2011; Murdoch et al. 1999; Ozkara et al. 2007; Persson et al. 2010; Schou et al. 2013; Shah et al. 2011; Stienen et al. 2018; Troughton et al. 2000). The remainder of the trials combined patients with preserved and reduced ejection fraction (Anguita et al. 2010; Berger et al. 2010; Carubelli et al. 2016; Eurlings et al. 2010; Lainchbury et al. 2009; Maeder et al. 2013; Pfisterer et al. 2009).

**Table 1:** Characteristics of included clinical trials

Clinical trial	Patients	Intervention arm (*n*) vs standard care arm (*n*)†	Follow-up (months)
Murdoch et al. (1999)	Stable CHF, LVEF ≤ 35%	BNP arm *n* = 10 Standard care *n* = 10	2
Troughton et al. (2000)	Decompensated HF now stabilised, LVEF< 40%	BNP arm *n* = 33 Standard care *n* = 36	9.5
Beck-da-Silva et al. (2005)	> 18 years, stable CHF but not on β blockers, LVEF ≤ 40%	BNP arm *n* = 21 Standard care *n* = 20	3
Jourdain et al. (2007)	> 18 years, optimized on treatment, LVEF < 45%	BNP arm *n* = 110 Standard care *n* = 110	15
Ozkara et al. (2007)	Treated with ACEI/loop diuretic, LVEF ≤ 50%	NT-proBNP arm *n* = 79‡ Standard care *n* = 61	6
Pfisterer et al. (2009)	≥ 60 years, LVEF≤v45%, 60–74 years=NT-proBNP ≥ 400 pg/ml; ≥ 75years = NT-proBNP 800 pg/ml	NT-proBNP arm *n* = 251 Standard care *n* = 248	18
Lainchbury et al. (2009)*	> 18 years, AHF now stabilised	NT-proBNP arm *n* = 121 Standard care *n* = 122	36
Anguita et al. (2010)	> 18 years, AHF	BNP arm *n* = 30 Standard care *n* = 30	18
Persson et al. (2010)	LVEF < 50%, males NT-proBNP > 800 ng/ml, females> 1000 ng/ml	NT-proBNP arm *n* = 125 Standard care *n* = 127	9
Eurlings et al. (2010)	AHF NT-proBNP > 1700, randomized at discharge if > 10% drop in NT-proBNP	NT-proBNP arm *n* = 174 Standard care *n* = 171	24
Berger et al. (2010)*	AHF now stabilised, LVEF < 40%	NT-proBNP + MC arm (only patients with NT-proBNP > 2200 pg/ml) *n* = 92 Standard care *n* = 90	Maximum 18; minimum 12
Januzzi Jr et al. (2011)	> 21 years, LVEF < 40%	NT-proBNP arm *n* = 75 Standard care *n* = 76	10
Shah et al. (2011)	Decompensation HF now stabilized, LVEF ≤ 35%	BNP arm *n* = 68 Standard care *n* = 69	4
Karlstrom (2011)	> 18 years; BNP > 150 ng/L for those aged < 75 years, and BNP > 300 ng/L for those aged > 75 years	BNP arm *n* = 147 Standard care *n* = 132	33
Maeder et al. (2013)	≥ 60 years, LVEF > 45%, 60–74 years = NT-proBNP ≥ 400 pg/ml; ≥ 75 years = NT-proBNP 800pg/ml	NT-proBNP arm *n* = 59 Standard care *n* = 64	18
Schou et al. (2013)	> 18years, Optimised on treatment and implantable ICD/CRT, LVEF < 45%, NT-proBNP > 1000	NT-proBNP arm *n* = 199 Standard care *n* = 208	Median 30
Carubelli et al. (2016)	Randomized after stabilization of AHF	NT-proBNP arm *n* = 137 Standard care *n* = 134	Mean 18
Stienen et al. (2018)	Decompensated HF, NT-proBNP levels > 1700 ng/ml within 24 h of hospital admission. In hospital intervention	NT-proBNP arm *n* = 201 Standard care *n* = 203	6
Felker et al. (2017)	LVEF ≤ 40%, NT-proBNP > 2000 pg/mL/BNP > 400 pg/ml	NT-proBNP arm *n* = 446 Standard care *n* = 448	12

*CHF* chronic heart failure, *AHF-*acute heart failure, *NT-proBNP* N-terminal pro b-type natriuretic peptide, *LVEF* left ventricular ejection fraction, *ARB* angiotensin II receptor blocker, *ACEI* angiotensin converting enzyme inhibitor, *ARA* aldosterone receptor antagonist, *B-blocker* beta blocker, *ICD/CRT* implantable converter defibrillator/cardiac resynchronisation therapy, *BNP* B-type natriuretic peptide, *MC* multidisciplinary care, *NYHA* New York Heart Association, *HF* heart failure

†Check Additional file 1

*Randomised to three-arm but only 2 meet the inclusion criteria for this review, NP-directed arm and control arm most reflecting usual patient care

‡ Only patients in the intervention arm received spironolactone

The conduct of the trial intervention arms is shown in Table 2. All trials randomized patients into NP-directed medical therapy or clinical/usual care. Two trials were three-arm trials, but for this analysis, only the interventional and usual care arms were included (Berger et al. 2010; Lainchbury et al. 2009). In the majority of trials, the NP threshold for inclusion was consistent across age and gender, with the exception of three trials, where the threshold was either age- or gender-specific (Karlstrom et al. 2011; Maeder et al. 2013; Persson et al. 2010; Pfisterer et al. 2009). Nine trials set population NP targets (Anguita et al. 2010; Berger et al. 2010; Carubelli et al. 2016; Felker et al. 2017; Januzzi et al. 2011; Jourdain et al. 2007; Lainchbury et al. 2009; Murdoch et al. 1999; Troughton et al. 2000), eight trials set individualized NP targets (Beck-da-Silva et al. 2005; Eurlings et al. 2010; Karlstrom et al. 2011; Maeder et al. 2013; Persson et al. 2010; Pfisterer et al. 2009; Schou et al. 2013; Shah et al. 2011; Stienen et al. 2018), and one trial had no set NP target, but directed medical therapy to reduce the NP level (Ozkara et al. 2007). The management of the standard care arms is shown in Additional file 1.

**Table 2:** The conduct of the natriuretic-peptide (NP)-directed clinical trials

Clinical Trial	Level of care in interventional group	Frequency of visits	NP target
Murdoch	Specialist HF clinic	Every 2 weeks	Single target BNP< 50 pg/ml
Troughton	Specialist HF clinic	Every 3 months	Single target N-BNP < 200 pmol/L
Beck-da- Silva	Nurse-led HF clinic	Every 3 months	Individualized according to symptoms in relation to BNP levels.
Jourdain	Specialist care at the clinic	1 month (for 3 months) then 3 months	Single target BNP < 100 pg/ml
Ozkara	Physician clinic visits	Treatment not adjusted throughout study	No BNP target set
Lainchbury *	Research clinic (with possible specialist input)	Every 3 months	Single target NT-proBNP < 150 pmol/L
Maeder; Pfisterer	Outpatients visits	1, 3, 6, 12, 18 months	NT-proBNP < 400 pg/ml in < 75 years and < 800 pg/ml in ≥ 75 years
Eurlings	Specialist care at the clinic	2 weeks, 1 month, then 3 months	Individualized NT-proBNP < 10% of randomization level
Berger *	HF specialist clinic	Every 2 weeks till NT-proBNP target met. Then as required.	Single target NT-proBNP < 2200 pg/ml
Persson	Primary care centres	10 days, 1, 3, 6, 9 months	Individualized NT-proBNP < 50% from baseline level
Anguita	Cardiology clinic	1, 2, 3, 6, 24, 18 months	Single target BNP < 100 pg/ml
Shah	HF clinic with specialist input	1 week, 1, 2, 3, 4 months after discharge	Individualized BNP < 2 times discharge level
Januzzi	HF clinic	Every 3 months	Single target NT-proBNP ≤ 1000 pg/ml
Karlstrom	Outpatient visits	2, 6, 10, 16, 2, 36, 48 weeks, then every 6 months	< 75 years (BNP < 15 ng/L) and ≥75yrs (BNP < 300 ng/L)
Schou	Specialist heart failure clinic	Every 1–3 months	Individualised NT-proBNP < 30% of randomization level
Carubelli	Single center, initially in hospital management and then outpatient visits	Frequent visits if NT-proBNP still raised after discharge. Then telephonic follow up at 1, 3, and 6 months	Single target NT-proBNP≤ 3000 pg/ml
Felker	Outpatient visits	2 and 6 weeks, then every 3 months	Single target NT-proBNP < 1000 pg/mL.
Stienen	Intervention carried out in the hospital	1 week and at 1, 3, and 6 months	Individualized to reduce NT-proBNP by at least 30% by discharge

*NP* natriuretic peptide, *NT-proBNP* N-terminal pro B-type natriuretic peptide, *LVEF* left ventricular ejection fraction, *ARB* angiotensin II receptor blocker, *ACE*Iangiotensin-converting enzyme inhibitor, *BNP* B-type natriuretic peptide, *NYHA* New York Heart Association, *HF* heart failure

*Lainchbury and Berger: three-arm trial but only NT-proBNP guided management group and usual care group compared

Two trials were stopped early (Felker et al. 2017; Januzzi et al. 2011), Felker et. al for the benefit, and Januzzi et al. for futility.

### Risk of bias within studies and across studies

The risk of bias of the included trials is shown in Additional file 1: Figures S1 and S2. The random sequence generation was unclear in half the trials, and blinding of patients and investigators was low. Many trials did not clearly document if outcome assessors were blinded. The funnel plots for SMD (Additional file 1: Figure S3), and 6-month mortality (Additional file 1: Figure S4) did not suggest publication bias.

### Results of individual studies and synthesis of results

#### The efficacy of an NP-directed medical therapy in reducing NP levels within 6 months compared to standard care

**Fig. 2.**
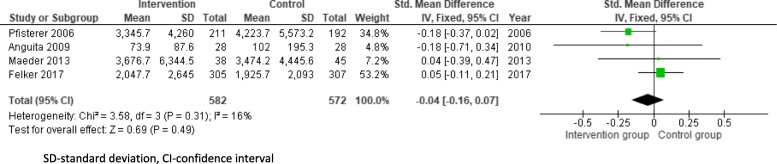
Efficacy of natriuretic peptide-directed medical therapy versus control in reducing BNP-levels within 6 months (Standardised mean difference in natriuretic peptide levels in NP-directed medical therapy clinical trials). SD, standard deviation; CI, confidence interval

Fourteen out of 18 medical therapy trials presented data on change in NP levels during the trial (Anguita et al. 2010; Carubelli et al. 2016; Eurlings et al. 2010; Felker et al. 2017; Januzzi et al. 2011; Jourdain et al. 2007; Karlstrom et al. 2011; Lainchbury et al. 2009; Maeder et al. 2013; Murdoch et al. 1999; Persson et al. 2010; Pfisterer et al. 2009; Schou et al. 2013; Shah et al. 2011; Stienen et al. 2018; Troughton et al. 2000), of which 7 out of 14 trials presented data on NP levels within the first 6 months of the trial (Anguita et al. 2010; Carubelli et al. 2016; Felker et al. 2017; Maeder et al. 2013; Pfisterer et al. 2009; Shah et al. 2011; Stienen et al. 2018). Three trials Shah, Carubelli, and Stienen were excluded as the data was reported at differing time points before 6 months: Stienen (mean 12 ± 10 days) (Stienen et al. 2018), Carubelli (mean 11 ± 9 days) (Carubelli et al. 2016) and Shah (4 months) (Shah et al. 2011). The overall point estimate of the four remaining trials was nonsignificant at 6 months of NP-directed medical therapy with low heterogeneity in the included trials (Fig. 2), (SMD − 0.04, 95% CI − 0.16, 0.07).

#### Reduction in NP levels and its association with mortality

Seventeen out of 18 studies reported mortality at trial completion (Anguita et al. 2010; Beck-da-Silva et al. 2005; Berger et al. 2010; Carubelli et al. 2016; Eurlings et al. 2010; Felker et al. 2017; Januzzi et al. 2011; Jourdain et al. 2007; Karlstrom et al. 2011; Lainchbury et al. 2009; Maeder et al. 2013; Ozkara et al. 2007; Persson et al. 2010; Pfisterer et al. 2009; Schou et al. 2013; Shah et al. 2011; Stienen et al. 2018; Troughton et al. 2000). After extracting the end of trial mortality data, it was deemed inappropriate to conduct a meta-analysis, as the duration of the trial follow up periods differed between the trials. It was therefore impossible to conduct a meta-analysis at a fixed long-term time point.

**Fig. 3:**
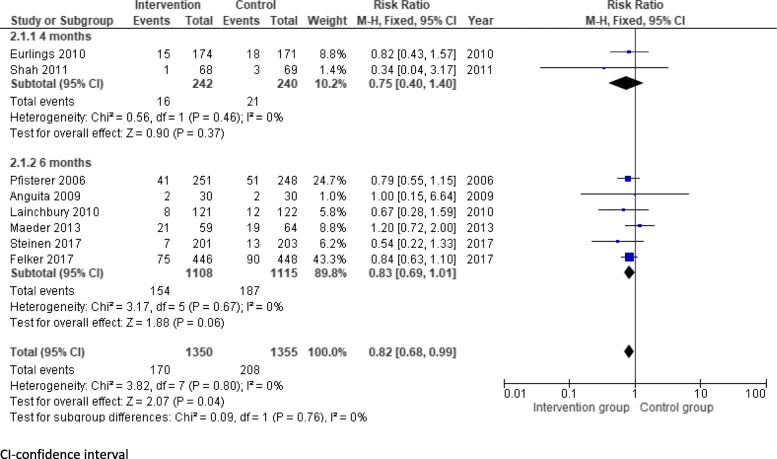
Effect of natriuretic peptide guided medical therapy versus control on mortality after 6 months. CI-confidence interval

Eight of the 18 trials (Anguita et al. 2010; Eurlings et al. 2010; Felker et al. 2017; Lainchbury et al. 2009; Maeder et al. 2013; Pfisterer et al. 2009; Shah et al. 2011; Stienen et al. 2018) reported mortality within the first 6 months of the intervention. Two trials reported mortality at 4 months, and 6 trials reported mortality at 6 months. NP-directed medical therapy was associated with a reduction in mortality within the first 6 months of the intervention (RR 0.82, 95% CI 0.68–0.99). Subgroup analysis suggested little heterogeneity between the 4-month and 6-month outcomes (Fig. 3).

#### Adverse events in relation to the change in therapy

Seven out of 18 studies recorded adverse effects of medical therapy on electrolytes and the cardiovascular system (Eurlings et al. 2010; Januzzi et al. 2011; Jourdain et al. 2007; Maeder et al. 2013; Murdoch et al. 1999; Pfisterer et al. 2009; Shah et al. 2011; Troughton et al. 2000). These were deemed not serious and six of these studies showed no difference in the incidence of adverse effects of therapy between the intervention and control groups.

#### The specific treatment strategies used in the trials

The treatment strategies and efficacy of these treatments varied between the trials. The two trials that showed the most benefit associated with NP-directed medical therapy,(Carubelli et al. 2016) and (Shah et al. 2011), showed efficacy for diuretics (the former) and angiotensin-converting enzyme inhibitors and beta-blockers (the latter). The two studies that showed the greatest number of patients reaching target NP levels, (Lainchbury et al. 2009) and (Karlstrom et al. 2011), showed that a combination of therapies was effective, including diuretics, angiotensin-converting enzyme inhibitor/angiotensin II receptor blockers and beta-blockers.

## Discussion

The principal findings of this systematic review are that NP-directed medical therapy does not significantly reduce NP levels at 6 months after initiation of NP-directed medical therapy. However, NP-directed medical therapy may be associated with decreased mortality in the short term, and there is little heterogeneity for this finding.

### Strengths

The strength of this review is that we evaluated the efficacy of NP-directed medical therapy from clinical trials, on the biochemical response of patient NP levels, and the clinically relevant outcome of mortality. The methodology of this systematic review and meta-analysis is robust.

### Findings in relation to other studies

There is an important fundamental difference between this meta-analysis, and the other two meta-analyses that were published after the protocol for our meta-analysis was registered (Khan et al. 2018; Pufulete et al. 2017). The primary outcome of our meta-analysis was to evaluate if it was possible to decrease NP levels with NP-directed therapy, while the primary outcome of the other two meta-analyses was to determine if NP-directed medical therapy was associated with a survival benefit. Evaluation of a potential survival benefit was a secondary outcome in our meta-analysis. Our primary interest was to determine whether perioperative physicians could possibly decrease NP levels prior to elective surgery in patients with high NP levels (and thereby potentially improve the risk profile of poor surgical candidates). Both these meta-analyses also had point estimates favoring survival benefit with NP-directed therapy in the long term. The importance of our meta-analysis is that (i) a reduction in NP levels is not necessarily essential to demonstrate a survival benefit with NP-directed medical therapy, and (ii) that this survival benefit may be seen earlier than what has been previously documented. The utility of NP-directed medical therapy in preoperative surgical patients is unknown, as there are currently no surgical trials in this field. This meta-analysis suggests that there is potential utility in this approach in surgical patients.

Preoperative risk stratification of high-risk patients is advocated by international guidelines, the most recent being the Canadian Cardiovascular Society Guidelines on Perioperative Cardiac Risk Assessment and Management for Patients Who Undergo Noncardiac Surgery (Duceppe et al. 2017). Screening for natriuretic peptides is a key component of risk stratification (Duceppe et al. 2017).

Our meta-analysis suggests that there may be a further benefit to the reduction of NP levels prior to surgery. The survival benefit seen with NP-directed medical therapy in this meta-analysis may suggest that an intensification of medical therapy is warranted in patients with marked physiological derangement reflected by a markedly elevated NP level. These high NP levels may reflect some reversibility in volume status and myocyte ischemia which is responsive to further medical therapy. Indeed, the trials which demonstrated the greatest number of patients reaching a target NP level included a combination of therapies which would have had both volume and ischemia efficacy (Karlstrom et al. 2011; Lainchbury et al. 2009). The importance of this systematic review is the following. Firstly, these findings suggest that there is potential to improve survival for an elective surgical population through NP-directed medical therapy. Secondly, the perioperative period is a powerful modifier of risk, and decreasing this risk, has the potential to change morbidity and mortality up to a year after surgery (Puelacher et al. 2018).

### Limitations

We were unable to obtain data from all the included trials for the SMD, the patients reaching the target NP and the time to NP reduction analysis. This is because most trials did not publish these end-points, nor was this included as part of the trial protocols. It is possible that if we had a larger sample which included data from all trials, then we may have shown an association between NP-directed medical therapy and a reduction in NP levels. However, it appears from this meta-analysis, that it is the intensification of medical therapy, rather than the reduction in NP levels, which may be important for short-term survival.

The non-parametric data for the SMD analysis was transformed to mean and standard deviation to facilitate analysis and caution should be taken when interpreting these results. The range of starting NP level on randomization in the intervention groups is large and could dramatically influence responsiveness to NP-directed therapy. However, despite these differences in the pre-intervention NP levels, mortality decreased in the NP-directed therapy arm, with little heterogeneity. It could be argued, however, that this early mortality (i.e., at 6 months of therapy initiation) signal is fragile. If a random-effects meta-analysis is conducted, then one cannot demonstrate a survival benefit associated with NP-directed medical therapy (RR 0.88, 95% 0.75–1.04, *p* = 0.14). Similarly, a sensitivity analysis which excludes all trials with a high risk of bias is not associated with a survival benefit (RR 0.84, 95% CI 0.61–1.15, *p* = 0.27) in a random-effects model. The survival benefit demonstrated in this meta-analysis therefore should be considered “hypothesis-generating” at best. It was not possible to control for the effect of age or renal function on NP for this analysis.

Finally, the included trials had very different implemented protocols, and thus it is not possible to identify a preferred medical management plan based on these data.

### Future research

This systematic review provides support for a clinical trial of preoperative NP-directed medical therapy in high-risk elective surgical patients.

## Conclusion

NP-directed medical therapy does not necessarily decrease NP levels, but it may be associated with a survival benefit. There may be a place for preoperative NP-directed medical therapy in high-risk surgical patients.

## Supplementary information

**Additional file 1: **Example of search strategy for the systematic review. Description of the standard care arm. **Table S1.** Data extracted for meta-analyses SMD – standardised mean difference, NP – natriuretic peptide. **Table S2.** AMSTAR evaluation of previous systematic reviews. **Figure S1.** Risk of bias summary. **Figure S2.** Risk of bias graph. **Figure S3.** Funnel plot for Standard Mean Difference forest plot. **Figure S4.** Funnel plot for mortality at 4 and 6 months forest plot.

## Data Availability

All articles available online and datasets are available from the corresponding author.

## Abbreviations

ACEI: Angiotensin-converting enzyme inhibitor
ARB: Angiotensin II receptor blocker
BB: Beta-blocker
BNP: B-type natriuretic peptide
CI: Confidence interval
IQR: Interquartile range
MINS: Myocardial injury after non-cardiac surgery
NP: Natriuretic peptides
NT-proBNP: N-terminal pro B-type natriuretic peptide
RR: Relative risk
SD: Standard deviation
SMD: Standardized mean difference
