# Supplementary material for: Natriuretic peptide-directed medical therapy: a systematic review
Source: Perioper Med (Lond). 2020 Feb 18;9:5. doi: 10.1186/s13741-019-0134-y (PMC7027249; doi:10.1186/s13741-019-0134-y)
Supplement: Supplementary file 1 — Additional file 1: Example of search strategy for the systematic review. Description of the standard care arm. Table S1. Data extracted for meta-analyses SMD – standardised mean difference, NP – natriuretic peptide. Table S2. AMSTAR evaluation of previous systematic reviews. Figure S1. Risk of bias summary. Figure S2. Risk of bias graph. Figure S3. Funnel plot for Standard Mean Difference forest plot. Figure S4. Funnel plot for mortality at 4 and 6 months forest plot. [file 13741_2019_134_MOESM1_ESM.rtf]

Supplementary Appendix 
Example of search strategy for the systematic review
Search strategy: (peptide set AND therapy set) NOT surgery set
Natriuretic Peptide, Brain (MeSH) OR
Brain Natriuretic Peptide OR Type-B Natriuretic Peptide OR Type B Natriuretic Peptide OR B-type Ventricular Natriuretic Peptide OR B type Ventricular Natriuretic Peptide
AND
Therapeutics (MeSH)
Therapeutics OR treatment OR therapy OR rehabilitation
NOT
General Surgery (MeSH) OR Surgical Procedures, Operative (MeSH) OR
surgery OR surgical OR operative
search strategy: (peptide set AND heart failure set) NOT surgery set
Natriuretic Peptide, Brain (MeSH) OR
Brain Natriuretic Peptide OR Type-B Natriuretic Peptide OR Type B Natriuretic Peptide OR B-type Ventricular Natriuretic Peptide OR B type Ventricular Natriuretic Peptide
AND 	
Heart Failure (MeSH) OR Coronary Artery Disease (MeSH)
Heart failure OR cardiac failure OR heart decompensation OR myocardial infarction OR myocardial ischemia OR heart attack OR myocardial infarct
NOT
General Surgery (MeSH) OR Surgical Procedures, Operative (MeSH) OR
surgery OR surgical OR operative
search strategy: (peptide set AND exercise set) NOT surgery set
Natriuretic Peptide, Brain (MeSH) OR
Brain Natriuretic Peptide OR Type-B Natriuretic Peptide OR Type B Natriuretic Peptide OR B-type Ventricular Natriuretic Peptide OR B type Ventricular Natriuretic Peptide
AND
Exercise (MeSH) OR Physical Exertion (MeSH) OR 
Exercise OR physical exertion OR physical activity OR motor activity OR physical effort
NOT
General Surgery (MeSH) OR Surgical Procedures, Operative (MeSH) OR
surgery OR surgical OR operative


Description of the standard care arm:
Anguita - Symptom-guided treatment to a clinical Framingham score <2
Carubelli - no further changes to medical therapy 
Felker - Follow 2013 ACC/AHA guidelines
Steinen - no further changes to therapy
Shah - Congestion score (indicative of fluid overload) done at patient discharge and diuretics titrated 
                  in these patients. 
Pfisterer and Maeder - Symptom guided treatment, reduce symptoms to dyspnea NYHA class of II or less.


Supplementary Appendix Table 1. Data extracted for meta-analyses SMD – standardised mean difference, NP – natriuretic peptide
Author	Year	SMD at 6 months	Mortality at 4 and 6 months	
Lainchbury	2010			
Eurlings	2010			
Shah	2011			
Pfisterer	2006			
Maeder	2013 			
Anguita	2009			
Felker	2017			
Stienen	2017			


Supplementary Appendix Table 2. AMSTAR evaluation of previous systematic reviews
Author	Journal	Comment	A piori design	Duplicate	Comprehensive review	Publication status	List of studies	Characteristics of
studies	Scientific quality assessed	Quality with conclusions	Publication bias discussed	
Cardarelli and Lamicao	JABFP
2003	Narrative
review 	No	No	No	No	Yes	No	No	No	No	
Porapakkham	Arch Intern Med 2010	Systematic review and  meta-analysis	No	No	No	No	Yes	Yes	No	Yes	Yes	
Li	Heart, Lung and Circulation 2013	Meta-analysis	No	Yes	No	No	Yes	Yes	Yes	Yes	No	
Savarese	PLOS ONE 2013	Systematic review and individual patient meta-analysis	Yes	Yes	No	No	No	No	No	Yes	Yes	
De Vecchis	Journal Cardiovascular Medicine 2014	Systematic review and meta-analysis	No	Yes	Yes	Yes	Yes	Yes	No	No	No	
Troughton	European Heart Journal 2014	Systematic review and individual patient meta-analysis	Yes	Yes	No	No	Yes	Yes	No	No	No	
Brunner-La Rocca	European Journal of Heart Failure 2015	Systematic review and individual patient meta-analysis	No	Yes	No	No	Yes	Yes	No	No	No	
Xin	Heart Fail Rev 2015	Systematic review and  meta-analysis	Yes	Yes	No	No	Yes	Yes	No	No	Yes	
Pufulete
	Systematic Reviews 2017	Systematic review and individual patient meta-analysis	Yes	Yes	Yes	Yes	Yes	Yes	Yes	Yes	Yes	
Khan	International Journal of Cardiology 2018	Systematic review and meta-analysis	No	Yes	Yes	Yes	Yes	Yes	Yes	Yes	No	


Supplementary Appendix Figure 1. Risk of bias summary.


Supplementary Appendix Figure 2. Risk of bias graph.


Supplementary Appendix Figure 3 Funnel plot for Standard Mean Difference forest plot


Supplementary Appendix Figure 4. Funnel plot for mortality at 4 and 6 months forest plot


References:
